# Satellite DNA as a Driver of Population Divergence in the Red Flour Beetle *Tribolium castaneum*

**DOI:** 10.1093/gbe/evu280

**Published:** 2014-12-19

**Authors:** Isidoro Feliciello, Ivana Akrap, Josip Brajković, Ivo Zlatar, Đurđica Ugarković

**Affiliations:** ^1^Department of Molecular Biology, Ruđer Bošković Institute, Zagreb, Croatia; ^2^Laboratory of Experimental Biology, Department of Clinical Medicine and Surgery, University of Naples Federico II, Italy

**Keywords:** satellite DNA, repetitive DNA, genome evolution, heterochromatin, population divergence, *Tribolium castaneum*

## Abstract

Tandemly repeated satellite DNAs are among most rapidly evolving sequences in eukaryotic genome, usually differing significantly among closely related species. By inducing changes in heterochromatin and/or centromere, satellite DNAs are expected to drive population and species divergence. However, despite high evolutionary dynamics, divergence of satellite DNA profiles at the level of natural population which precedes and possibly triggers speciation process is not readily detected. Here, we characterize minor TCAST2 satellite DNA of the red flour beetle *Tribolium castaneum* and follow its dynamics among wild-type strains originating from diverse geographic locations. The investigation revealed presence of three distinct subfamilies of TCAST2 satellite DNA which differ in monomer size, genome organization, and subfamily specific mutations. Subfamilies Tcast2a and Tcast2b are tandemly arranged within pericentromeric heterochromatin whereas Tcast2c is preferentially dispersed within euchromatin of all chromosomes. Among strains, TCAST2 subfamilies are conserved in sequence but exhibit a significant content variability. This results in overrepresentation or almost complete absence of particular subfamily in some strains and enables discrimination between strains. It is proposed that homologous recombination, probably stimulated by environmental stress, is responsible for the emergence of TCAST2 satellite subfamilies, their copy number variation and dispersion within genome. The results represent the first evidence for the existence of population-specific satellite DNA profiles. Partial organization of TCAST2 satellite DNA in the form of single repeats dispersed within euchromatin additionally contributes to the genome divergence at the population level.

## Introduction

Comparison of genomic structure and organization among populations or closely related species that are still hybridizing can reveal the loci and regions with elevated divergence. Such loci might act as drivers of population divergence and could be important for the potential speciation process. Among most rapidly evolving sequences in eukaryotes are satellite DNAs, tandemly repeated sequences assembled within heterochromatin at the (peri)centromeric and subtelomeric regions. Satellite DNA can exist in the form of varieties known as subfamilies, and each subfamily is represented by a specific repetitive sequence. Both, the assortment of the satellite sequences and/or the relative amount of satellite DNAs can vary between closely related species representing a sort of “satellite DNA species-specific content.” Even if two subfamilies located in the same genome are highly similar, they maintain the individual subfamily specific primary structure and exhibit the high homogeneity of repeats known as concerted evolution (Feliciello et al*.* 2005, 2006, 2011). Several examples raise the possibility that satellite DNA repeats themselves can act as drivers of population and species divergence by inducing changes in heterochromatin and/or centromere and playing a role in speciation (Ugarković and Plohl 2002). Pericentromeric regions rich in satellite DNA show fixed differences between incipient species of mosquito *Anopheles gambiae* in contrast with other regions of genome where many polymorphisms are shared, and are proposed to act as speciation islands (White et al. 2010). Comparison between sequenced and assembled genomes of two naturally hybridizing avian species of flycatchers revealed repetitive satellite elements at centromeres and telomeres as genomic regions with the highest level of divergence (Ellegren et al. 2012). In addition, it was shown that species-specific heterochromatin block composed of 359 satellite repeats present on *Drosophila melanogaster* X chromosome, but absent on *Drosophila simulans* X chromosome specifically prevents mitotic chromosome segregation causing lethality in F1 daughters coming from *D. melanogaster* males and *D. simulans* females (Ferree and Barbash 2009). It can be envisaged that due to the lack of adequate proteins and/or noncoding RNAs heterochromatin can become a barrier for crossing between two related species or even populations (Brown et al*.* 2012; Ferree and Prasad 2012). According to the meiotic drive model of speciation a copy number change of centromeric satellite DNA sequences which is followed by adaptive evolution of satellite DNA-binding proteins leads to rapid centromere evolution (Malik and Henikoff 2009). In maize, two types of tandemly repeated satellite sequences are shown to be involved in meiotic drive, and by mediating chromosome mobility they could proliferate and possibly trigger speciation (Kanizay et al*.* 2013). All these examples indicate that changes in satellite DNA sequence and/or content could in some cases influence evolution of species.

Although divergence of satellite DNA profiles can be detected between closely related species, it is still not clear if divergence already occurs at the population level and precedes speciation. Satellite DNA divergence at the population level analyzed in different insect species such as cave cricket *Dolichopoda schiavazzi* (Bachmann et al*.* 1994), leaf beetle *Xanthogaleruca luteola* (Lorite et al*.* 2002), *Drosophila buzzatti* species cluster (Kuhn et al*.* 2003, Kuhn and Sene 2004; de Franco et al*.* 2006) as well as in pinewood nematode *Bursaphelenchus xylophilus* (Vieira et al*.* 2014) revealed no sequence characteristics or other features that could discriminate a population or groups of populations. Only in satellite DNAs of pupfish (Elder and Turner 1994) divergence of satellite DNAs characterized by fixation of population-specific mutations was detected, whereas in the grasshopper *Eyprepocnemis plorans* satellite DNA showed variation in chromosomal distribution among populations (Cabrero et al*.* 2003). Insect genus *Tribolium* (Tenebrionidae, Coleoptera) is characterized by the presence of large blocks of pericentromeric heterochromatin composed almost exclusively of satellite DNAs which differ significantly among species and form species-specific profiles (Ugarković et al. 1996). *Tribolium castaneum* has a major satellite DNA TCAST1 which comprises 35% of the whole genome (Feliciello et al. 2011) and encompasses centromeric as well as pericentromeric regions of all 20 chromosomes (Ugarković et al*.* 1996). Study of TCAST1 satellite DNA dynamics at the level of *T. castaneum* natural populations revealed low differences in mutational profiles, but no significant difference in the monomer size, organization, and copy number was detected (Feliciello et al*.* 2011).

In order to obtain more comprehensive insight into the potential influence of satellite DNA on genome divergence between *T. castaneum* populations and consequently on speciation process, we decided to characterize a minor satellite DNA TCAST2 and to follow its dynamics among wild-type strains originating from diverse geographic locations. The investigation revealed presence of distinct subfamilies of TCAST2 satellite DNA which differ in monomer size, genomic location, and exhibit subfamily specific mutations. In addition, TCAST2 subfamilies are differentially amplified among *T. castaneum* strains resulting in copy number variation which enables discrimination between strains. This is the first evidence for the existence of population-specific satellite DNA profiles and strong support for the hypothesis that satellite DNA can act as driver of genome divergence at the population level. Partial dispersion of TCAST2 satellite DNA within euchromatin additionally contributes to the genome divergence at the population level.

## Materials and Methods

### Beetle Strains and Genomic DNA Extraction

The following strains of *T. castaneum* were used: GA2 strain, originally used in the genome sequencing project and deriving from North American wild-type strain collected in Georgia in 1982, obtained from Dr Dick Beeman (Manhattan, KS) and wild-type strains: GA1, collected in Georgia, USA in 1980; 43, collected at Kyushu Island, Japan in 1988; 50, collected in Schegel Farm, Indiana, USA in 2005; 51, collected in Adrian, Missouri, USA in 2006; 52, collected in Bloomington, Indiana, USA in 2006; 55, collected in Jerez, Spain in 1991; 57, collected in Perù, in 2002; 61, collected in Banos, Ecuador in 1996; Zg, collected near Zagreb (Božjakovina), Croatia in 2010; and VT, collected at Veliko Trgovišće, Croatia in 2010. Each laboratory stock was established and maintained as a separate culture at a population size of greater than 200 individuals on standard medium (20:1, flour:brewer’s yeast, by weight) in a dark incubator at 24 °C and approximately 70% relative humidity. DNA was extracted from 50 to 100 mg of adult insects, which corresponds to 10–20 individuals, following the instruction of DNeasy Blood & Tissue Kit (Qiagen) for high molecular weight DNA purification.

### Primers Designed for Polymerase Chain Reaction Amplification

TCAST2 satellite was amplified using two sets of primers: 2ab amplifies both Tcast2a and Tcast2b repeats (forward: 2ab-F, 5′-GTTTATCATCCACGAGGCG-3′; reverse: 2ab-R, 5′-CACTCGTTTTCACTCGTGG-3′) and 2a (forward: 2a-F, 5′-CCGTAAATTTTGACAGTGTCG-3′; reverse: 2a-R, 5′-CGGTTTTTCTCCTGTGTAAGG-3′) was designed from the region present only in Tcast2a and not in Tcast2b. Primers P1 (5′-ATAATCTGACGTTTCGTACTG-3′) and P2 (5′-CTTTAAGTGACATTTTCAAATG-3′) were used to amplify part of Tcast2a repeat. Primers for amplification of dispersed element present in the first intron of gene annotated as 663838 were: forward 5′ ACTGGCTATAACCGTCCACC3′ and reverse 5′-GCTGGACTTCAGGAAGAGCT3′.

### Polymerase Chain Reaction Amplification

Polymerase chain reactions (PCRs) reactions were performed separately for each strain in a 25 µl containing 50 ng of template DNA, 1 × PCR buffer, 2 mM of MgCl_2_, 0.2 mM of each dNTP, 0.2 µM of each primer, and 0.02 U/µl of Taq DNA polymerase (Fermentas). PCR was performed under the following conditions: 3 min denaturation at 94 °C, 35 cycles of 30 s at 94 °C, 30 s at 54 °C, and 1 min at 72 °C, and a final extension of 3 min at 72 °C. PCR amplification products were subjected to 1.2% (w/v) agarose gel electrophoresis and stained with ethidium bromide. A specific touchdown PCR program was designed to amplify multimers: 3 min denaturation at 94 °C, 15 cycles of 30 s at 94 °C, 30 s at 57–0.25 °C and 1 min at 72 °C, 25 cycles of 30 s at 94 °C, 30 s at 52 °C, and 1 min at 72 °C, and a final extension of 3 min at 72 °C.

### Genomic Sequencing

The PCR amplification products obtained after using both primer sets with DNA of each strain were excised from the agarose gels using the QIAquick PCR Purification Kit (Qiagen, Germany). For each strain a band of approximately 360 bp and/or of approximately 180 bp were obtained using the 2ab primer set, and a band of approximately 360 bp was obtained with the 2a primer set. The direct and inverse sequence electropherograms of each amplified repeat were compared and the genomic sequences were converted into a genomic consensus sequence using IUBMB single letter code for multiple bases in the same position (e.g., Y = C + T, K = T + G, W = A + T, S = G + C, R = A + G, M = A + C, D = A + T + G, H = A + T + C). The most common sequence (MCS) was obtained by indicating the predominant base found at each variable position. PCR was performed under the following conditions: 5 min denaturation at 95 °C, 30 cycles of 1 min at 95 °C, 1 min at 54 °C, and 1 min at 72 °C, and a final extension of 3 min at 72 °C. The sequencing was performed using the automatic sequencer ABI Prism 310 (Applied Biosystems).

### Fluorescent In Situ Hybridization

Chromosome spreads were prepared from male gonads as described in Ugarković et al*.* (1996). Fluorescent in situ hybridization (FISH) was performed with 20 ng/μl of biotinylated Tcast2a (360 bp) probe obtained by primers 2ab or Tcast2a-specific probe prepared by P1-P2 primers. Biotinylated probes were made by PCR, using bio-16-dUTP (Roche): 95 °C for 3 min, 30 cycles at 94 °C for 30 s, 52 °C for 30 s, 70 °C for 1 min, and 2 min at 72 °C. Hybridization was performed for 20 h at 37 °C in a solution composed of 60% formamide, 2 × SSC, 10% dextran sulfate, and 20 mM sodium phosphate. Posthybridization washes were done at 37 °C: two times in 50% formamide/2 × SSC and two times in 4 × SSC/0.1%Tween20/1%BSA. The samples were stained with 5 ug/ml fluorescein avidin D and biotinylated antiavidin D (Vector laboratories). The chromosomes were counterstained with DAPI Nucleic Acid Stain (Invitrogen) and analyzed with a Zeiss Axiovert 35 inverted stage fluorescent microscope equipped with a Pixera digital camera system.

### Southern and Dot-Blot Hybridization

Southern blot analysis was performed under high stringency conditions as described previously (Feliciello et al*.* 2011). Primers 2ab were used to prepare a biotinylated probe using as a template purified Tcast2a (360 bp) or Tcast2b (179 bp) repeat amplified by PCR from genomic DNA. Results obtained with the two types of probes were equivalent. For quantitative dot-blot analysis DNA samples at 1 mg/ml in denaturing buffer (0.4M NaOH, 1M NaCl) were serially diluted with an equal volume of herring sperm DNA (Promega) at the same concentration of 1 mg/ml in denaturing buffer. A 200 µl aliquot of each dilution was added per slot in a dot-blot apparatus (Bio-Rad), filtered on a nylon membrane Nytran N (Amersham Hybond—GE Healthcare) and fixed on a membrane by baking at 80 °C. As a standard, a purified sample of a specific PCR-amplified Tcast2a and Tcast2b from genomic DNA was used at an initial concentration of 3 ng. Hybridization and staining procedures were the same as those used for Southern blot analysis. Biotinylated probe is a Tcast2a specific subunit obtained using primers P1 and 2aRev.

### Quantitative Real-Time PCR

Quantitative PCR (qPCR) reactions were carried out in a total volume of 50 µl consisting of 1 µl DNA (final concentration 10 ng/µl), 25 µl 2 × Power SYBR Green PCR Master Mix (Applied biosystems), and 200 nM of each primer, P1, and 2aRev. For normalization single copy gene WD-repeat containing protein 47 (accession number: 657535) was used. Primers for this gene are F1 (5′-TTCTCTGTACCCTTCGAGCAA3′) and R1 (5′-ATTGGAGTGACGAGCATGTG3′). qPCR reactions were run in triplicate on an ABI 7300 instrument. The thermal cycling conditions were as follows: 50 °C for 2 min, 95 °C for 7 min, 95 °C for 15 s, and 60 °C for 1 min for 40 cycles followed by dissociation stage: 95 °C for 15 s, 60 °C for 1 min, 95 °C for 15 s, and 60 °C for 15 s. Amplification specificity was confirmed by dissociation curve analysis. Specificity of amplified product was additionally tested on agarose gel. No template control was included in each run. Postrun data were analyzed using LinRegPCR software v. 11.1 (Ruijter et al*.* 2009, 2013). “*N*_0_ value” determined for each technical replicate was averaged. Averaged *N*_0_ values for TCAST2 reactions were divided by *N*_0_ values of endogenous control for normalization.

### Bioinformatic Search for TCAST2 Elements and Phylogenetic Analysis

The NCBI refseq_genomic database of *T. castaneum* and all scaffolds that have not been mapped to linkage groups were screened using BLASTN version 2.2.22 + . The program was optimized to search for highly similar sequences (megablast) to the query sequence—TCAST2a consensus sequence and blast hits on the query sequence were analyzed individually. Sequences corresponding to hits were analyzed by dot plot (http://www.vivo.colostate.edu/molkit/dnadot/), using standard parameters (window size 9, mismatch limit 0), or more relaxed conditions (window size 11, mismatch limit 1), to determine the exact start and end site of specific TCAST2 element. Repbase, a reference database of eukaryotic repetitive DNA, was screened using WU-BLAST (Kohany et al*.* 2006).

Sequence alignment was performed using MUSCLE algorithm (Edgar 2004) combined with manual adjustment. Gblocks was used to eliminate poorly aligned positions and divergent regions of the alignments (Talavera and Castresana 2007). Maximum-likelihood (ML) trees were estimated with the PhyML 3.0 software (Guindon and Gascuel 2003) whereas Markov chain Monte Carlo Bayesian searches were performed in MrBayes v. 3.1.2. (Huelsenbeck and Ronquist 2001). Branch support was evaluated by bootstrap analysis (1,000 replicates) in ML and by posterior probabilities in Bayesian analyses. Pairwise sequence diversity (uncorrected *p*) was calculated using the MEGA 5.05 software (Tamura et al*.* 2011).

## Results

### Identification and Characterization of TCAST2 Satellite DNA Divergence among Populations

**Fig. 1.—:**
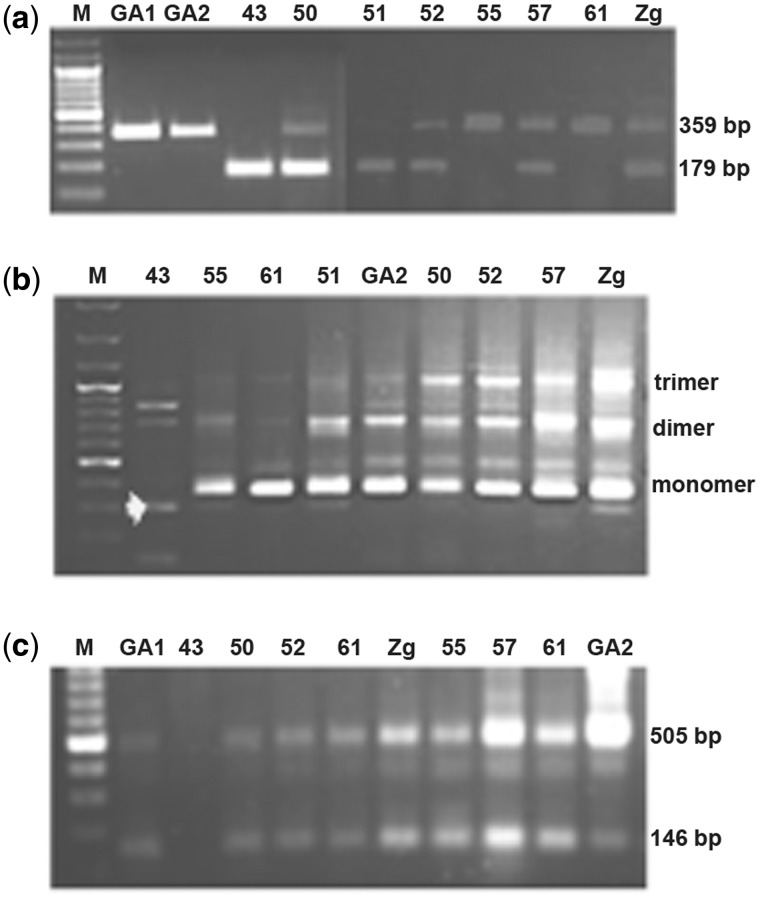
PCR amplification of TCAST2 satellite DNA from different *T. castaneum* populations using pairs of primers: (*a*) 2ab, (*b*) 2a (position of a 300 bp fragment obtained after amplification of DNA from strain 43 is indicated by white arrow), and (***c***) P1P2.

Although the genome sequencing project of *T. castaneum* has been completed (Richards et al. 2008), most of the heterochromatic regions that are prevalently composed of satellite DNA were excluded from the genome sequence due to technical difficulties associated with sequencing and assembling highly repetitive regions, and the number of clones that contain tandemly arranged repeats is very limited in the database. Bioinformatic analysis of *T. castaneum* genome revealed presence of highly repetitive families and each of them occupies more than 0.1% of the genome (Wang et al*.* 2008). We decided to check experimentally if these repetitive DNAs might belong to the tandemly arranged satellite DNAs. Among them, the repetitive DNA identified by Wang et al. (2008) as *R* = 20, of approximately 300 bp, was used to design a pair of primers named 2ab, starting from the same position of the repetitive unit but in the opposite orientation which enables amplification of tandem repeats. PCR amplification was done on genomic DNA from ten *T. castaneum* strains which originate from diverse geographic locations in North- and South America, Europe and Japan, characterized by distinct environments ranging from continental and humid climate, Mediterranean climate to subtropical and tropical climate. Two main amplification products having sizes of approximately 360 and 180 bp were obtained from DNA samples of strains 50, 51, 52, 57, and 61, one band of 360 bp was present in samples GA1, GA2, 55, and 61, whereas only a band of 180 bp was detected in strain 43 (fig. 1*a*). The results indicate presence of two subfamilies of repetitive family named TCAST2 which differ in monomer size and in distribution among strains.

**Fig. 2.—:**
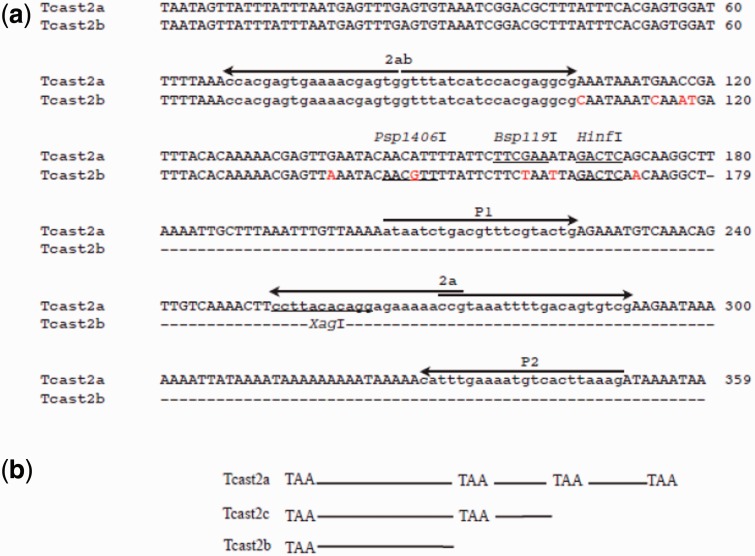
(*a*) The MCSs of Tcast2a and Tcast2b subfamilies in the species *T. castaneum.* Sequence alignment shows a high similarity except for the nine base substitutions indicated in red. Position of pairs of PCR primers 2ab, 2a, and P1P2 is shown and recognition sites for the restriction enzymes *Hinf*I, *Bsp*119I, *Psp*1406I, and *Xag*I are underlined. (*b*) Schematic representation of TCAST2 satellite subfamilies Tcast2a, Tcast2c, and Tcast2b. Positions of TAA motifs located around positions 1, 180, 300, and 360 nt of Tcast2a subfamily, which could be responsible for intrastrand homologous recombination and creation of subfamilies Tcast2c and Tcast2b, are indicated.

The 360 and 180 bp amplification products deriving from all ten strains were directly sequenced. The same direct and reverse primers used for PCR amplification were used for sequencing and obtained electropherograms showed clear prevalence of a single, particular base at most of the positions, indicating high sequence homogeneity of repeats within both amplification products. To simplify the comparative sequence analysis between different strains, an MCS was obtained by indicating the predominant base, if present, found at each variable position. Supplementary figure S1, Supplementary Material online, illustrates the MCSs of two TCAST2 subfamilies from different strains: Tcast2a and Tcast2b with 359 and 179 bp monomers, respectively. The results indicated an extremely high homogeneity of each subfamily within as well as between different strains. Figure 2*a* shows the MCSs of Tcast2a and Tcast2b subfamilies in the species *T. castaneum.* Sequence alignment of the two subfamilies showed a high similarity with a common region limited to 179 bp, and it is clear that Tcast2a and Tcast2b belong to the same satellite family. Furthermore and probably most interestingly, all Tcast2b monomers have 9 bp-specific variations with respect to Tcast2a, even in strains that contain both subfamilies, like strains 50 and 52.

From Tcast2a and Tcast2b sequences, we were able to design primers’ pair 2a which is supposed to be specific for Tcast2a subfamily. Also in this case the primers start from the same sequence origin but with opposite direction, as indicated in figure 2*a*. The PCR program was set to amplify multimers and the amplification products showed a clear organization of Tcast2a in tandem repeats (fig. 1*b*). A monomer, dimer, and trimer bands are visible on agarose gel and are present in all strains tested except in 43 (Japan) strain, where faint bands are amplified but with different size unrelated to Tcast2a. The band from 43 strain corresponding to about 300 bp was excised from the gel and the sequence showed to be highly similar to the region of TCAST2, as indicated schematically in figure 2*b*, containing 23 and 21 base substitutions with respect to the MCSs of Tcast2a and Tcast2b, respectively (supplementary fig. S2, Supplementary Material online). This result demonstrated the presence of a third TCAST2 subfamily termed Tcast2c, present not only in strain 43 but in other tested strains (fig. 1*b*). Based on the experimental results, TCAST2 satellite DNA seems to be organized in three subfamilies: Tcast2a and Tcast2b, generally excluded from the genome sequence assembly because of the inability to assemble tandem repeats and with probable location in constitutive heterochromatin, and Tcast2c which can be also detected by computational analysis (see next section) due to the dispersion within euchromatin.

In order to demonstrate a different allocation and organization of satellite DNA between strains, we decided to confirm the absence of Tcast2a subfamily in the strain 43 from Japan (fig. 1*a* and *b*). We designed a perfect Tcast2a specific-primer from the region ranging from 300 to 359 nt, indicated as P2 (position of primer is indicated on fig. 2*a*). This primer was used in a combination with P1 forward primer starting from 204 nt position (fig. 2*a*), giving a PCR amplification product of 146 bp (fig. 1*c*). The experiment, repeated three times using 40 pooled individuals of insects each time, indicated clearly the absence of Tcast2a in the strain 43 from Japan (fig. 1*c*, lane 2) whereas in other strains two main bands of 146 and 505 bp were present, as expected (fig. 1*c*). The band of 505 bp can be amplified only if Tcast2a is organized at least as a dimer, supporting the already detected tandem organization of Tcast2a.

### Detection and Analysis of Dispersed TCAST2 Elements

**Fig. 3.—:**
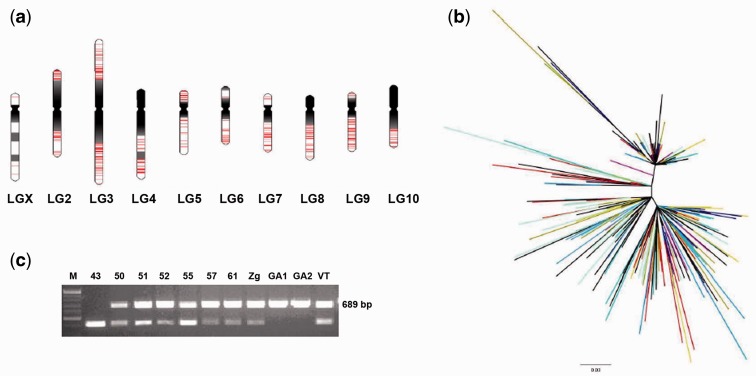
(*a*) Distribution of dispersed TCAST2 elements shown in red, on *T. castaneum* chromosomes. The karyotype representing the haploid set of *T. castaneum* chromosomes, and positions of constitutive heterochromatin (dark) and euchromatin (white) are depicted, based on C-banding data (Stuart and Mocelin 1995) and *T. castaneum* 3.0 assembly (www.beetlebase.org). Two TCAST2 elements are represented as separate lines if they are at least 100 kb distant from each other. (*b*) ML phylogenetic tree of dispersed TCAST2 elements. Elements are shown in different colors depending on the chromosome from which they originate: ch1, dark green; ch2, light green; ch3, black; ch4, purple; ch5, turquoise; ch6, yellow; ch7, dark blue; ch8, ochre; ch9, red; ch 10, pink. Bootstrap values are not indicated because they do not exceed 80%. (*c*) PCR amplification of a single dispersed TCAST2 element located within intron of a protein-coding gene (accession number 663838) on chromosome 2, in different *T. castaneum* populations.

We screened the NCBI refseq_genomic database of *T. castaneum*, using the alignment program BLASTN version 2.2.22 + in order to find elements similar to the experimentally determined 359 bp consensus sequence of Tcast2a satellite DNA. Only genomic sequences with at least 50 nt of continuous sequence, and greater than 80% identity to the Tcast2a consensus sequence were considered for further analysis. The search revealed total of significant hits, 367 of them found within the assembled *T. castaneum* genome dispersed on all chromosomes ranging from 3 on chromosome 1(X) to 99 on chromosome 3 (supplementary table S1, Supplementary Material online), while eight elements are located in the genome scaffolds that have not been mapped to the linkage groups. The positions of dispersed TCAST2-similar elements are specifically indicated on the haploid set of chromosomes (fig. 3*a*) based on the position within the genomic sequence, while regions of constitutive heterochromatin and euchromatin were assigned based on C-banding data (Stuart and Mocelin 1995) and *T. castaneum* 3.0 Assembly data. The size of the elements is in the range between 50 and 371 bp whereas very few of them have full size of 359 bp of Tcast2a consensus sequence determined experimentally. Seventy percent of elements are approximately 300 bp long, they show high sequence similarity to Tcast2a element starting from nucleotide 1 to 3 and ending at nt 299 to 304, which is a region of overlapping between Tcast2a and Tcast2c (fig. 2*b*, supplementary fig. S2, Supplementary Material online). They also share a high sequence similarity and monomer length with Tcast2c subfamily, and because of that are considered as members of Tcast2c subfamily. The remaining dispersed elements are shorter and truncated at 5′- and/or 3′-ends. Only one element is composed of two tandemly arranged monomers 296 and 301 bp long, while in all other cases the elements are dispersed in the form of single monomers or their parts. Different from experimentally detected Tcast2a and Tcast2b subfamilies which exhibit tandem organization, Tcast2c subfamily seems to be preferentially organized in dispersed form.

To test if there is any clustering of dispersed TCAST2 satellite sequences which could suggest presence of chromosome-specific groups, the alignment and subsequent phylogenetic analysis was performed. Because dispersed TCAST2 elements differ significantly in size, the alignment and phylogenetic analyses was performed on 307 elements that mutually overlap in their sequences whereas the others were excluded from the analysis. The alignments were additionally adjusted using Gblocks (supplementary file S1, Supplementary Material online). The average pairwise divergence among dispersed TCAST2 elements is 11.0%. Phylogenetic tree obtained by ML method (fig. 3*b*) revealed a very weak resolution of dispersed TCAST2 elements with no bootstrap values which exceed 80%. The tree shows no significant clustering of elements derived from the same array or the same chromosome. Bayesian method gave tree of similar topology and weak resolution (data not shown).

To check if dispersed TCAST2 elements show insertion polymorphism among strains, we analyzed several loci using PCR primers designed immediately up- and downstream of the TCAST2 insertions, based on sequence data of GA2 strain. PCR amplification of a single dispersed TCAST2 element located within intron of a protein-coding gene (accession number 663838) on chromosome 2 in different *T. castaneum* strains gave products corresponding to 389 and 689 bp (fig. 3*c*) which were subsequently sequenced (supplementary fig. S3, Supplementary Material online). The results reveal 300 bp TCAST2c element inserted into both alleles in strains GA1 and GA2, in seven strains it is inserted in a single allele, whereas strain 43 has no insertion. Although more comprehensive analysis of dispersed loci is necessary, this preliminary data indicate that dispersed TCAST2 elements also contribute to genome divergence among *T. castaneum* strains.

### Organization of TCAST2 Subfamilies Using Fluorescent In Situ and Southern Hybridization

**Fig. 4.—:**
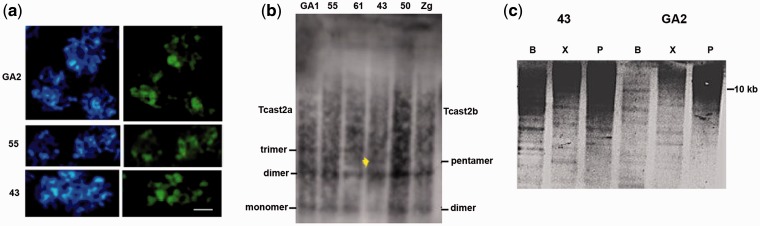
(*a*) Sets of *T. castaneum* chromosomes from populations GA2, 55, and 43, in meiotic prometaphase, after staining with fluorochrome DAPI (left) and after FISH using as a probe TCAST2 satellite DNA (right). The bright green fluorescent signals, representing position of satellite DNA, are colocalized with heterochromatic blocks. The bar represents 5 μm. (*b*) Southern hybridization with a probe specific for TCAST2 satellite on DNA from different populations of *T. castaneum* digested with restriction enzymes *Hinf*I (in line 43 position of pentamer of Tcast2b subfamily is indicated with yellow arrow). (*c*) Southern hybridization on DNA from populations 43 and GA2 digested with *Bsp*119I (lines B), *Psp*1406I (lines P), and *Xag*I (lines X), after hybridization with TCAST2 probe.

Using PCR experiments and bioinformatic analysis, we detected presence of tandemly arranged 359 bp Tcast2a and 179 bp Tcast2b elements as well as of 300 bp Tcast2c elements present preferentially in dispersed form in *T. castaneum* genome. To study organization of these satellite subfamilies on *T. castaneum* chromosomes and genomic DNA, we used hybridization experiments with a probe corresponding to Tcast2a monomer which hybridizes with all three TCAST2 subfamilies. FISH on chromosomes from strains GA2 and 55 which have Tcast2a subfamily, as well as strain 43 with Tcast2b subfamily, revealed strong signals which coincide with the regions of pericentromeric heterochromatin on all chromosomes which are in the meiotic pachytene phase (fig. 4*a*). This indicates that tandemly repeated Tcast2a and Tcast2b elements, respectively, are located within the same regions of pericentromeric heterochromatin. Small dots within euchromatic tails might indicate dispersed Tcast2c elements, although the presence of Tcast2c elements within pericentromeric heterochromatin cannot be also excluded. No hybridization signal was detected on chromosomes from strain 43 using probe specific for Tcast2a subfamily (data not shown) indicating absence of this subfamily in 43 strain.

Southern hybridization was performed on DNAs from strains GA1, 55, and 61 characterized by the presence of Tcast2a subfamily, strain 43 containing Tcast2b subfamily and strains 50 and Zg having both Tcast2a and Tcast2b subfamilies. DNAs were digested with restriction enzyme *Hinf*I which has single recognition site in all three TCAST2 subfamilies (recognition site is indicated on fig. 2*a*). The hybridization, using the Tcast2a probe common to all three subfamilies, revealed a ladder with prominent bands of approximately 350 and 700 bp which can correspond to Tcast2a monomer/dimer and Tcast2b dimer/tetramer in all digestions (fig. 4*b*). Higher bands within a ladder are detectable as well as an intermediate band of approximately 900 bp in 43 strain which could correspond to Tcast2b 5-mer. Due to the significant presence of dispersed TCAST2 elements, in particular Tcast2c subfamily, there is a strong background signal on Southern blot which prevents resolution and disables fine distinction between patterns of different strains. However, Southern blot confirms a tandem repeat organization of TCAST2. Southern hybridization was also performed using the restriction enzymes *Bsp1*19I and *Xag*I which are supposed to preferentially cut Tcast2a subfamily and enzyme *Psp*1406I which specifically cuts within Tcast2b (fig. 2*a*). As expected, hybridization analysis reveals more extensive digestion with enzymes *Bsp*119I and *XagI* on DNA from GA2 strain which predominantly contains Tcast2a subfamily, relative to DNA from strain 43 characterized by the predominance of Tcast2b subfamily (fig. 4*c*). Hybridization pattern obtained on *Psp*1406I digested DNA from GA2 and 43 strains reveals relatively weak digestion of both GA2 and 43 strains DNA. Most probably this result may be a consequence of TCAST2 DNA methylation considering that *Psp*1406I is methylation sensitive restriction enzyme and our previous results showed methylation of TCAST1 satellite DNA (Feliciello et al*.* 2013). Although the complex pattern of digestion with all three enzymes is not easy to interpret, it is consistent with the presence of different TCAST2 subfamilies in GA2 and 43 strains.

### Quantification of TCAST2a in *T. castaneum* Strains by Dot Blot and Real Time qPCR

**Fig. 5.—:**
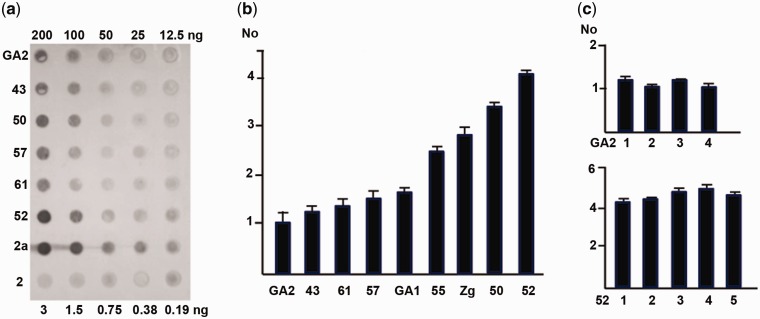
Quantification of copy number of Tcast2a satellite subfamily in different populations of *T. castaneum*. (*a*) Dot-blot hybridization with the amounts of DNA indicated on the blot. As a positive control Tcast2a DNA in row 2a was spotted in amounts indicated on blot. The same amounts of Tcast2b DNA were spotted in row 2 as a negative control. (*b*) qPCR on DNAs from different populations. No values represent the mean values of two independent experiments performed in triplicate and error bars represent standard deviations. (*c*) Tcast2a copy number variation within populations GA2 and 52 revealed by qPCR. Different DNA isolations (columns 1–4 and 1–5, respectively) each from 40 individuals, were tested. The experiments were performed in triplicate and error bars represent standard deviations.

A reduction or increase of a satellite DNA copy number may influence heterochromatin structure and represents an important and underestimated source of genome variation (Ugarković and Plohl 2002). Our results reveal different allocation of two satellite DNA subfamilies among *T. castaneum* strains: Tcast2a is present in all strains except one (43) whereas Tcast2b was not detected in four strains. This indicates that copy number of the satellite DNA changes very fast and can be monitored at the population level. To demonstrate a copy number variability of TCAST2 satellite DNA between strains, we performed dot-blot analysis using as hybridization probe the region amplified by P1-forward/2a-reverse primers pair (fig. 2*a*). This probe recognizes Tcast2a subfamily, but can also hybridize with Tcast2c repeats. The intensity of hybridization signals was measured by densitometry, and the relative amounts of the satellite subfamily in the genomic DNAs were determined using the calibration curve based on signals of diluted Tcast2a satellite monomers. Hybridization reveals that Tcast2a satellite DNA content is variable among strains of *T. castaneum* and corresponds from 0.38% to 0.75% of the total genomic DNA (fig. 5*a*). Considering genome size of *T. castaneum* which is 0.21 pg or 204 Mb (Alvarez-Fuster et al*.* 1991), monomer size of Tcast2a and calculated percentage of Tcast2a within genomic DNAs from different strains, copy number of Tcast2a is calculated. The results show that the Tcast2a + Tcast2c repeats are present in about 2,000 copies in some strains (GA2, 43, 57, and 61) and in about 4,000 copies in strains like 50 and 52, per haploid genome.

To confirm these results and also for a more precise evaluation of intraspecific variability of satellite DNA copy number, the quantification experiment was repeated by real time qPCR using the same P1-forward/2a-reverse primer pair. The results showed significant difference in copy number among nine strains with the largest difference of four times between 52 and GA2 (fig. 5*b*). The results are in accordance with those of dot blot showing the highest copy number in strains 52 and 50, the lowest in GA2, 43, 57, 61, and GA1, while 55 and Zg exhibit intermediate number of copies (fig. 5*b*). We performed qPCR using different DNA samples, isolated from a total of 160–200 individuals from strains 52 and GA2, respectively, to assess intrapopulation variability of TCAST2 copy number (fig. 5*c*). The results did not show significant individual variation in copy number within two tested strains. However dot blot and qPCR experiments confirm high dynamics in TCAST2 satellite copy number variation among strains.

## Discussion

Numerous studies of satellite DNA divergence at the population level did not succeed to identify population-specific mutations or other population-specific sequence features of satellite DNAs (Bachmann et al*.* 1994, Lorite et al*.* 2002, Kuhn et al*.* 2003, Vieira et al*.* 2014). Because all of these studies were based on the analysis of a relatively limited number of cloned repeats that did not represent the whole population of repeats within a genome, it is possible that slight changes in the mutational profiles remained hidden. Direct sequencing of PCR amplicons of a satellite DNA seems to represent a more suitable approach to study overall satellite DNA variability, as demonstrated previously for S1 satellite DNA in frogs (Picariello et al*.* 2002, Feliciello et al*.* 2005), *T. castaneum* major satellite DNA TCAST1 (Feliciello et al*.* 2011) and here for TCAST2 satellite. Genomic consensus sequences of TCAST2 satellite revealed presence of two highly similar subfamilies: Tcast2a with 359 bp monomer and Tcast2b which corresponds in sequence to the first 179 bp of Tcast2a monomer (fig. 2*b*). The sequences of two subfamilies are distinguished by nine point mutations, each homogenized and fixed within a particular subfamily. Sequences of satellite subfamilies do not differ among *T. castaneum* strains but subfamilies are differentially amplified among strains: four strains have Tcast2a subfamily, a single strain has Tcast2b, whereas five strains reveal parallel presence of both subfamilies.

TCAST2 satellite DNA seems to follow the “library” model of evolution which explains the difference in satellite DNA profiles among closely related species (Ugarković and Plohl 2002) and was experimentally confirmed in different coleopteran genera (Meštrović et al*.* 1998; Pons et al*.* 2004). According to this model the creation of species-specific satellite profiles occurs by differential amplification of satellite DNAs shared among related species (Ugarković and Plohl 2002). In the case of *T. castaneum* strains, differential amplification of TCAST2 subfamilies occurs and results in population-specific satellite profiles. Differential amplification can occur due to genetic and environmental factors which affect heterochromatin structure and consequently lead to the instability and rearrangement of tandem repeats (Pezer and Ugarković 2008). Among genetic factors, unequal crossingover (Ma and Jackson 2006) and rolling circle replication of extrachromosomal circular DNA (Assum et al*.* 1993; Navrátilová et al*.* 2008) might induce continuous change of satellite DNA amount and heterochromatin content. Such changes are proposed to contribute to significant inter- and intraspecific heterogeneity of genome size observed in *Tribolium* and *Drosophila* species (Alvarez-Fuster et al*.* 1991; Bosco et al*.* 2007) and are characteristic for S1 satellite DNA of frog *Rana italica* (Cardone et al*.* 1997). The rolling circle amplification mechanism might be also responsible for the observed sequence homogeneity of TCAST2 satellite subfamilies. It was proposed that this mechanism might replace segments that have accumulated mutated repetitive units with new homogenous segments and in this way could act as satellite DNA repair mechanism (Feliciello et al*.* 2005, 2006). Indication in favor of the action of such mechanism is the higher divergence of dispersed TCAST2 elements relative to the tandem repeats within constitutive heterochromatin. Regarding environmental factors, *T. castaneum* heterochromatin is highly sensitive to heat stress which induces satellite DNA demethylation (Feliciello et al*.* 2013) and overexpression, accompanied by changes of heterochromatin-specific histone modifications (Pezer and Ugarković 2012). Such remodeling of heterochromatin structure could stimulate reorganization of satellite DNA arrays, leading to a relatively rapid change in a copy number of particular satellite or satellite subfamilies, or even their almost complete loss such as Tcast2a subfamily in strain 43. The final outcome of this process might be the creation of population-specific satellite DNA profiles, as observed in *T. castaneum*. What might be the biological consequence of the differential amplification and significant copy number variation of TCAST2 satellite subfamilies among strains? Crossing between individuals from strains GA2 and 43 resulted in no significant difference in the number of offspring in the first and second generation relative to the number of offspring within these strains (data not shown). This preliminary experiment indicates that difference in TCAST2 satellite profiles might not be involved in hybrid incompatibility between the two strains. TCAST2 is present in relatively low copy number in the genome and based on results of in situ hybridization, it seems to be dispersed within heterochromatin, among long arrays of a major and predominant TCAST1 satellite DNA. TCAST1 exhibits slight changes in mutation profiles among strains as well as conservation in copy number (Feliciello et al. 2011). It might be proposed that the conservation of TCAST1 is sufficient for heterochromatin preservation in interstrain crosses in spite of changes in TCAST2 profiles.

**Fig. 6.—:**
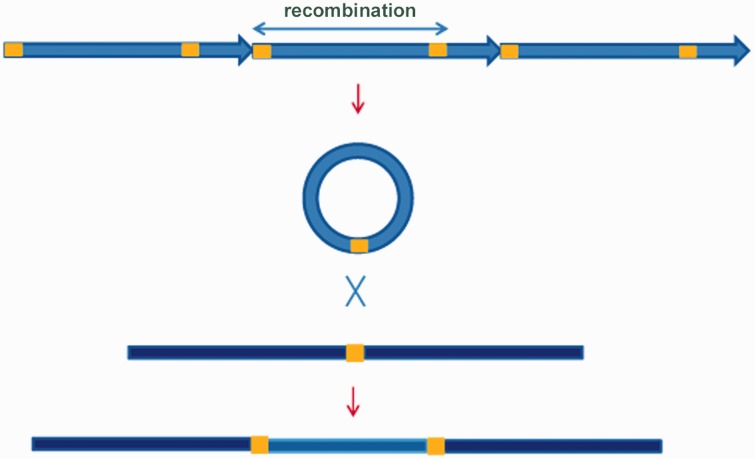
The mechanism of dispersion of TCAST2 elements throughout the genome based on the excision of satellite elements in the form of extrachromosomal circles due to the intrastrand homologous recombination between AT-rich motifs, represented by ochre rectangles. It is followed by insertion of TCAST2 elements into genome which is mediated by site directed recombination between homologous motifs in satellite elements and in target genomic sequence.

Besides tandemly arranged Tcast2a and Tcast2b subfamilies located within heterochromatin, experiments and bioinformatic analysis of *T. castaneum* genome revealed a presence of a third Tcast2c subfamily present in a dispersed form within euchromatin of all chromosomes. It is composed mostly of 300 bp single repeats similar to the heterochromatic subfamilies Tcast2a and Tcast2b. Tcast2c might be created by intrastrand recombination of Tcast2a element due to the homology of short AT-rich sequence motifs located at the beginning of Tcast2a and near position 300 (fig. 2*b*). The same mechanism of intrastrand recombination between homologous AT-rich motifs near start site and in the middle of Tcast2a element could explain the emergence of Tcast2b subfamily (fig. 2*b*). Analysis of flanking regions of dispersed Tcast2c elements revealed AT-rich sequence motifs, 5–9 nt long, duplicated at the sites of insertion. It can be proposed that due to intrastrand recombination within TCAST2 arrays extrachromosomal circles are formed. Site directed recombination then can occur between motifs within TCAST2 extrachromosomal circular DNAs and homologous motifs within genome, leading to dispersion of TCAST2 elements in the euchromatin (fig. 6). The majority of dispersed TCAST2 satellite DNA repeats is in the form of monomer probably due to higher rate of intra-molecular recombination events which occur in euchromatin with respect to heterochromatin. Therefore, homologous recombination, probably stimulated by environmental stress, seems to trigger the emergence of new satellite subfamilies and their dispersion throughout the genome. A part of TCAST2 element (159 bp) shares 70% similarity with DNA transposon Crypton-2_TCa deposited within Repbase (http://www.girinst.org/repbase/), indicating that this mobile element might have contributed to TCAST2 spreading throughout the genome.

Similar to TCAST2, major TCAST1 satellite DNA elements were also found to be distributed within euchromatin, in the vicinity of genes (Brajković et al*.* 2012). Intraspecies difference in the pattern of distribution of transposons as well as of tandem repeats is described for some plant populations and is proposed to be under the influence of stressful conditions (Kalendar et al*.* 2000; Raskina et al*.* 2011). In addition, dispersed transposable elements contribute to gene expression divergence between related plant species (Hollister et al*.* 2011). Our preliminary experiments show that dispersion patterns of TCAST1 and TCAST2 satellite elements vary among *T. castaneum* strains and if satellites perform gene regulatory role as proposed (Pezer et al*.* 2012, Brajković et al*.* 2012) then they might also contribute to the gene expression divergence at the population level. In conclusion, our investigation presents the evidence for the significant variation of satellite DNA profiles at the population level and suggests the potential influence of satellite DNA dynamics on evolution of species.

## Supplementary Material

Supplementary file S1, table S1, and figures S1–S3 are available at *Genome Biology and Evolution* online (http://www.gbe.oxfordjournals.org/).

Supplementary Data
