# Supplementary material for: Satellite DNA as a Driver of Population Divergence in the Red Flour Beetle Tribolium castaneum
Source: Genome Biol Evol. 2014 Dec 19;7(1):228–39. doi: 10.1093/gbe/evu280 (PMC4316633; doi:10.1093/gbe/evu280)
Supplement: Supplementary Data [file supp_7_1_228__index.html]

Satellite DNA as a driver of population divergence in the red flour beetle Tribolium castaneum — Satellite DNA as a Driver of Population Divergence in the Red Flour Beetle Tribolium castaneum — Supplementary Data 

# Satellite DNA as a Driver of Population Divergence in the Red Flour Beetle *Tribolium castaneum*

## Supplementary Data

files

**Files in this Data Supplement:**

- Supplementary Data - zip file
