# Supplementary material for: Satellite DNA as a Driver of Population Divergence in the Red Flour Beetle Tribolium castaneum
Source: Genome Biol Evol. 2014 Dec 19;7(1):228–39. doi: 10.1093/gbe/evu280 (PMC4316633; doi:10.1093/gbe/evu280)
Supplement: Supplementary Data [file supp_evu280_suppl_data.zip › New Microsoft Office Word Document.docx]

**Legends to supplementary data:**

**Fig. S1.** Alignment of the most common sequences of TCAST2a and TCAST2b subfamilies from ten *T. castaneum* strains. Nine base substitutions which distinguish sequence of TCAST2b from TCAST2a are indicated in red, as well as changes among TCAST2a sequences from different strains. Y= C+T, K=T+G, M=A+C.

**Fig. S2.** Alignment of the most common sequences of Tcast2a and Tcast2b subfamilies in *T. castaneum* with Tcast2c subfamily from strain 43. Base substitutions relative to the consensus sequence of all three subfamilies are indicated in red. TAA motifs are indicated in green.

**Fig. S3.** Alignment of TCAST2 element inserted within intron of gene with accession number 663838 and its flanking region in strain GA2 with the corresponding region in population 43. Inserted TCAST2 element is shown in brown and base substitutions which distinguish sequences of GA2 and 43 strains are shown in red.

**Table S1.** Number of dispersed TCAST2 elements on *T. castaneum* chromosomes.

**Supplementary file 1.** Alignment of dispersed TCAST2 elements adjusted using Gblocks which eliminates poorly aligned positions and divergent regions.
