# Supplementary material for: Satellite DNA as a Driver of Population Divergence in the Red Flour Beetle Tribolium castaneum
Source: Genome Biol Evol. 2014 Dec 19;7(1):228–39. doi: 10.1093/gbe/evu280 (PMC4316633; doi:10.1093/gbe/evu280)
Supplement: Supplementary Data [file supp_evu280_suppl_data.zip › suppl. figures revised.doc]

```
Figure S1

Tcast2a-strain52       TAATAGTTATTTATTTAATGAGTTTGAGTGKAAATCGGACGCTTTATTTCACGAGTGGAT 60
Tcast2a-strainZG       TAATAGTTATTTATTTAATGAGTTTGAGTGTAAATCGGACGCTTTATTTCACGAGTGGAT 60
Tcast2a-strain50       TAATAGTTATTTATTTAATGAGTTTGAGTGKAAATCGGACGCTTTATTTCACGAGTGGAT 60
Tcast2a-strainGA1      TAATAGTTATTTATTTAATKARTTTGARTGTAAATCGGACGCTTTATTTCACGAGTGGAT 60
Tcast2a-strainGA2      TAATAGTTATTTATTTAATGAGTTTGAGTGTAAATCGGACGCTTTATTTCACGAGTGGAT 60
Tcast2a-strain55       TAATAGTTATTTATTTAATGAGTTTGAGTGTAAATCGGACGCTTTATTTCACGAGTGGAT 60
Tcast2a-strain61       TAATAGTTATTTATTTAATGAGTTTGAGTGTAAATCGGACGCTTTATTTCACGAGTGGAT 60

Tcast2b-strain50       TAATAGTTATTTATTTAATGAGTTTGAGTGTAAATCGGACGCTTTATTTCACGAGTGGAT 60
Tcast2b-strain52       TAATAGTTATTTATTTAATGAGTTTGAGTGTAAATCGGACGCTTTATTTCACGAGTGGAT 60
Tcast2b-strain43       TAATAGTTATTTATTTAATGAGTTTGAGTGTAAATCGGACGCTTTATTTCACGAGTGGAT 60


Tcast2a-strain52       TTTTAAACCACGAGTGAAAACGAGTGGTTTATCATCCACGAGGCGAAATAAATGAACCGA 120
Tcast2a-strainZG       TTTTAAACCACGAGTGAAAACGAGTGGTTTATCATCCACGAGGCGAAATAAATGAACCGA 120
Tcast2a-strain50       TTTTAAACCACGAGTGAAAACGAGTGGTTTATCATCCACGAGGCGAAATAAATGAACCGA 120
Tcast2a-strainGA1      TTTTAAACCACGAGTGAAAACGAGTGGTTTATCATCCACGAGGCGAAATAAATGAACCGA 120
Tcast2a-strainGA2      TTTTAAACCACGAGTGAAAACGAGTGGTTTATCATCCACGAGGCGAAATAAATGAACCGA 120
Tcast2a-strain55       TTTTAAACCACGAGTGAAAACGAGTGGTTTATCATCCACGAGGCGAAATAAATGAACCGA 120
Tcast2a-strain61       TTTTAAACCACGAGTGAAAACGAGTGGTTTATCATCCACGAGGCGAAATAAATGAACCGA 120

Tcast2b-strain50       TTTTAAACCACGAGTGAAAACGAGTGGTTTATCATCCACGAGGCGCAATAAATCAAATGA 120
Tcast2b-strain52       TTTTAAACCACGAGTGAAAACGAGTGGTTTATCATCCACGAGGCGCAATAAATCAAATGA 120
Tcast2b-strain43       TTTTAAACCACGAGTGAAAACGAGTGGTTTATCATCCACGAGGCGCAATAAATCAAATGA 120


Tcast2a-strain52       TTTACACAAAAACGAGTTGAATACAACATTTTATTCTTCGAAATAGACTCAGCAAGGCTT 180
Tcast2a-strainZG       TTTACACAAAAACGAGTTGAATACAACATTTTATTCTTCGAAATAGACTCAGCAAGGCTT 180
Tcast2a-strain50       TTTACACAAAAACGAGTTGAATACAACATTTTATTCTTCGAAATAGACTCAGCAAGGCTT 180
Tcast2a-strainGA1      TTTACACAAAAACGAGTTGAATACAACATTTTATTCTTCGAAATAGACTCAGCAAGGCTT 180
Tcast2a-strainGA2      TTTACACAAAAACGAGTTGAATACAACATTTTATTCTTCGAAATAGACTCAGCAAGGCTT 180
Tcast2a-strain55       TTTACACAAAAACGAGTTGAATACAACATTTTATTCTTCGAAATAGACTCAGCAAGGCTT 180
Tcast2a-strain61       TTTACACAAAAACGAGTTGAATACAACATTTTATTCTTCKAAATAGACTCAGCAAGGCTT 180

Tcast2b-strain50       TTTACACAAAAACGAGTTAAATACAACGTTTTATTCTTCTAATTAGACTCAACAAGGCT- 179
Tcast2b-strain52       TTTACACAAAAACGAGTTAAATACAACGTTTTATTCTTCTAATTAGACTCAACAAGGCT- 179
Tcast2b-strain43       TTTACACAAAAACGAGTTAAATACAACGTTTTATTCTTCTAATTAGACTCAACAAGGCT- 179


Tcast2a-strain52       AAAATTGCTTTAAATTTGTTAAAAATAATCTGACGTTTCGTACTGAGAAATGTCAAACAG 240
Tcast2a-strainZG       AAAATTGCTTTAAATTTGTTAAAAATAATCTGACGTTTCGTACTGAGAAATGTCAAACAG 240
Tcast2a-strain50       AAAATTGCTTTAAATTTGTTAAAAATAATCTGACGTTTCGTACTGAGAAATGTCAAACAG 240
Tcast2a-strainGA1      AAAATTGCTTTAAATTTGTTAAAAATAATCTGACGTTTCGTACTGAGAAATGTCAAACAG 240
Tcast2a-strainGA2      AAAATTGCTTTAAATTTGTTAAAAATAATCTGACGTTTCGTACTGAGAAATGTCAAACAG 240
Tcast2a-strain55       MAAATTGCKTTAAATTTGTTAAAAATAATCTGACGTTTCGTACTGAGAAATGTCAAACAG 240
Tcast2a-strain61       AAAATTGCKTTAAATTTGTTAAAAATAATCTGACGTTTCGTACTGAGAAATGTCAAACAG 240

Tcast2b-strain50       ------------------------------------------------------------
Tcast2b-strain52       ------------------------------------------------------------
Tcast2b-strain43       ------------------------------------------------------------


Tcast2a-strain52       TTGTCAAAACTTCCTTACACAGGAGAAAAACCGTAAATTTTGACAGTGTCGAAGAATAAA 300
Tcast2a-strainZG       TTGTCAAAACTTCCTTACACAGGAGAAAAACCGTAAATTTTGACAGTGTCGAAGAATAAA 300
Tcast2a-strain50       TTGTCAAAACTTCCTTACACAGGAGAAAAACCGTAAATTTTGACAGTGTCGAAGAATAAA 300
Tcast2a-strainGA1      TTGTCAAAACTTCCTTACACAGGAGAAAAACCGTAAATTTTGACAGTGTCGAAGAATAAA 300
Tcast2a-strainGA2      TTGTCAAAACTTCCTTACACAGGAGAAAAACCGTAAATTTTGACAGTGTCGAAGAATAAA 300
Tcast2a-strain55       TTGTCAAAACTTCCTTACACAGGAGAAAAACCGTAAATTTTGACAGTGTCGAAGAATAAA 300
Tcast2a-strain61       TTGTCAAAACTTCCTTACACAGGAGAAAAACCGTAAATTTTGACAGTGTCGAAGAATAAA 300

Tcast2b-strain50       ------------------------------------------------------------
Tcast2b-strain52       ------------------------------------------------------------
Tcast2b-strain43       ------------------------------------------------------------


Tcast2a-strain52       AAAATTATAAAATAAAAAAAAATAAAAACATTTGAAAATGTCACTTAAAGATAAAATAA 359
Tcast2a-strainZG       AAAATTATAAAATAAAAAAAAATAAAAACATTTGAAAATGTCACTTAAAGATAAAATAA 359
Tcast2a-strain50       AAAATTATAAAATAAAAAAAAATAAAAACATTTGAAAATGTCACTTAAAGATAAAATAA 359
Tcast2a-strainGA1      AAAATTATAAAATAAAAAAAAATAAAAACATTTGAAAATGTYACTTAAAGATAAAATAA 359
Tcast2a-strainGA2      AAAATTATAAAATAAAAAAAAATAAAAACATTTGAAAATGTCACTTAAAGATAAAATAA 359
Tcast2a-strain55       AAAATTATGAAATAAAAAAAAATAAAAACATTTGAAAATGTCACTTAAAGATAAAATAA 359
Tcast2a-strain61       AAAATTATGAAATAAAAAAAAATAAAAACATTTGAAAATGTCACTTAAAGATAAAATAA 359

Tcast2b-strain50       -----------------------------------------------------------
Tcast2b-strain52       -----------------------------------------------------------
Tcast2a-strain43       -----------------------------------------------------------


Figure S2


Tcast2a      TAATAGTTATTTATTTAATGAGTTTGAGTGTAAATCGGACGCTTTATTTCACGAGTGGAT 60
Tcast2c(43)  TAATAGTTATTTATTCGACGAGTTTGAGTGTAAATCGGACGTCTTATTTCACGAGTGGTT 57
Tcast2b      TAATAGTTATTTATTTAATGAGTTTGAGTGTAAATCGGACGCTTTATTTCACGAGTGGAT 60

Tcast2a      TTTTAAACCACGAGTGAAAACGAGTGGTTTATCATCCACGAGGCGAAATAAATGAACCGA 120
Tcast2c(43)  TTTTATACCTCGGGTGATAACGAGAGGTTTACCATCCACGAGGTGAATTAAATGAACCGA 117
Tcast2b      TTTTAAACCACGAGTGAAAACGAGTGGTTTATCATCCACGAGGCGCAATAAATCAAATGA 120

Tcast2a      TTTACACAAAAACGAGTTGAATACAACATTTTATTCTTCGAAATAGACTCAGCAAGGCTT 180
Tcast2c(43)  TTTACACGAAAACAAGTTGAATACAACATTTTATTCTTCGAATTAGACTCAACAAGACTT 177
Tcast2b      TTTACACAAAAACGAGTTAAATACAACGTTTTATTCTTCTAATTAGACTCAACAAGGCT- 179

Tcast2a      AAAATTGCTTTAAATTTGTTAAAAATAATCTGACGTTTCGTACTGAGAAATGTCAAACAG 240
Tcast2c(43)  AAAATTGCTTAAAATCTATTAAAAATAATCTGACGTTTCGTATTGAAAAATGCCAAACAG 237
Tcast2b      ------------------------------------------------------------

                                    2a primers region
Tcast2a      TTGTCAAAACTTccttacacaggagaaaaaccgtaaattttgacagtgTCGAAGAATAAA 300
Tcast2c(43)  TTGTCAAAACTGccttacacaggagaaaaaccgtaaattttgacagtgTCGAAGAA---- 296
Tcast2b      ------------------------------------------------------------

Tcast2a      AAAATTATAAAATAAAAAAAAATAAAAACATTTGAAAATGTCACTTAAAGATAAAATAA  359
Tcast2c(43)  -----------------------------------------------------------
Tcast2b      -----------------------------------------------------------


Figure S3

GA2             ACTGGCTATAACCGTCCACCATTTTTTGCTATCGTCAGACTAAAACTGAGTTCTGTTATA
43              ACTGGCTATAACCGTCCACCATTTTTTGCTATCGTCAGACTAAAACTGAGTTCTGTTATA

GA2             GTCTGGGTTTGAAATAAATCTAGTTTTGGCAAATTAAAGTTAGAAACTAAATTTATATTT
43              GTCTGGGTTTGAAATAAATCTAGTTTTGGCAAATTAAAGTTAGCAACTAAATTTATATAA

GA2             TTTAAAAATTGTTGTAATTCATGTGAGTATGCACAGAAATTGAAATATTTGCATCTTGGT
43              TTTAAAAATTGTTGTAATTCATGTGAGTATGCACAGAAATTGAAATATTTGCATCTTGGT

GA2             CTTAGAAATGTGTGTCATTGGAGCATTTAATTAATTTGTTAATTACGAATAATTTGCAAT
43              CTTAGAAATGTATGTCATTGGAGCATTTAATTAATTTGTTAATTACGAATAATTTGCAAT

GA2             GACATAAATGTACAGCCTTAAATATCTTATTAAAATTAAACGAGAAG-AAAAAACCTTTAT
43              GACATAAATGTACAGCCTTAAATATCTTATTAAAATTAAACGAAAAGGAAAAAACCT---

GA2             TCTTCGACACTGTCAAAATTTACGGTTTTTCTCCTGTGTAAGGAAGTTTTGACAATTGTT
43              ------------------------------------------------------------

GA2             TGGCATTTCTCAGTACGAAACGTCAGATTATTTTTAACAAATTTAAAGCAATTTTAAGCC
43              ------------------------------------------------------------

GA2             TTGCTGAGTCTATTTCGAAGAATAAAATGTTGTATTCAACTCGTTTTTGTGTAAATCGGT
43              ------------------------------------------------------------

GA2             TCATTTATTTCGCCTCATGGATGATAAACCACTCGTTTTCACTCGTGGTTTAAAAATCCA
43              ------------------------------------------------------------

GA2             CTCGTGAAATAAAGCGTCCGATTTACACTCAAACTCATTAAATAAATAACTATTACGTAT
43              ----------------------------------------------------TTACGTAT

GA2             TTATAACGATAATGTTA--TTTTTTTATAATACTTACACATTCTTCTCAATTAGAGATAT
43              TTATAACGATAATGTTAATTTTTTTTATAATACTTACACATTCTTCTCATTTAGAGATAT

GA2             CTATCGAAAAAGCTCTTCCTGAAGTCCAGC
43              CTATCGAAAAAGCTCTTCCTGAAGTCCAGC
```

**Table S1: Number of dispersed TCAST2 elements/chromosome of *T. castaneum***

| **Chromosome**  **number** | **Number of**  **TCAST2 elements** |
| --- | --- |
| **1 (X)** | **3** |
| **2** | **46** |
| **3** | **99** |
| **4** | **33** |
| **5** | **31** |
| **6** | **23** |
| **7** | **31** |
| **8** | **33** |
| **9** | **48** |
| **10** | **20** |
